# Estimating the public economic gains in Taiwan from *in vitro* fertilization (IVF) subsidy changes implemented in 2021

**DOI:** 10.1093/humrep/deae271

**Published:** 2024-12-13

**Authors:** Mei-Jou Chen, Nikos Kotsopoulos, Amy Ming-Fang Yen, Kuan-Ting Lin, Mark P Connolly

**Affiliations:** Department of Obstetrics and Gynecology, National Taiwan University Hospital, Taipei, Taiwan; Health Economics, Global Market Access Solutions Sarl, St-Prex, Switzerland; Department of Economics (UoA MBA), University of Athens, Athens, Greece; School of Oral Hygiene, College of Oral Medicine, Taipei Medical University, Taipei, Taiwan; Department of Obstetrics and Gynecology, National Taiwan University Hospital, Taipei, Taiwan; Health Economics, Global Market Access Solutions Sarl, St-Prex, Switzerland; Global Health, University Medical Center Groningen (UMCG), Groningen, The Netherlands

**Keywords:** assisted reproduction, IVF, medically assisted reproduction, economics, public economics, infertility, public policy

## Abstract

**STUDY QUESTION:**

What is the governmental fiscal impact of a new assisted reproduction subsidy scheme based on projected lifetime net taxes attributed to resulting live births in Taiwan?

**SUMMARY ANSWER:**

We estimate that the new fertility reimbursement scheme has generated favorable lifetime fiscal gains for the Taiwanese government, resulting in a return on investment (ROI) of NT$5.6 for every NT$1.0 spent based on those families receiving public subsidies for fertility care under the new scheme.

**WHAT IS KNOWN ALREADY:**

Globally, there is variation in the amount of public reimbursement for assisted reproduction provided to infertile couples. Cost is an important consideration for many infertile couples that can influence the amount of services provided and the types of services used.

**STUDY DESIGN, SIZE, DURATION:**

The analysis is based on the number of live births resulting from those couples receiving public subsidies for assisted reproduction. The cohort is based on those children born between March 2022 and July 2023.

**PARTICIPANTS/MATERIALS, SETTING, METHODS:**

A lifetime fiscal model was developed to project age-specific lifetime tax revenue and age-dependent benefits likely received from government attributed to the children born. The analysis is based on age-specific projected earnings adjusted for work activity and applied to published income tax burden data, in addition to estimated indirect consumption taxes paid. Furthermore, we estimate the lifetime national insurance contributions per worker, including employer contributions. To account for changes over the modeling period, we increased wages based on historical economic growth, government benefits were increased based on the rate of consumer price inflation rate, and all costs and taxes were discounted at 3.5%.

**MAIN RESULTS AND THE ROLE OF CHANCE:**

A child born in Taiwan in 2022 is expected to pay discounted gross tax revenues of NT$7 257 438 and receive NT$5 373 730 in discounted future benefits from the government. Following implementation of the new funding policy, based on the number of resulting births, the cost per live birth is NT$331 918. Applying the cost per live birth, we estimate the discounted net tax revenue to be NT$1 551 789 for each child born from the subsidy. The ROI for the Taiwanese government is estimated at 568% over the lifetime of the IVF-conceived children.

**LIMITATIONS, REASONS FOR CAUTION:**

Several assumptions are applied in making long-term financial projections. Should economic conditions change dramatically, this could influence the projections described in our work.

**WIDER IMPLICATIONS OF THE FINDINGS:**

The results suggest the government benefits from public subsidy for fertility services when taking into consideration the long-term work activity of these children and future tax revenue generated for government. The results are broadly applicable to other markets, although variations in wages, lifetime work activity, and taxation rates would influence the conclusions reported here.

**STUDY FUNDING/COMPETING INTEREST(S):**

The work was sponsored by the Merck Group in Singapore (funding to N.K. and M.P.C.). The sponsoring organization was given an opportunity to review the final manuscript; however, the authors retained full editorial control over the final published materials. The authors hold no financial interests in the sponsoring company. N.K. and M.P.C. have received consulting fees from Merck and Organon and payment/honoraria from Merck. M.-J.C. received no funding for this work but has received honoraria for lectures from the Taiwanese Society for Reproduction Medicine, Taiwanese Association of Obstetrics and Gynecology, Japanese Society of Obstetrics and Gynecology, The Endocrine Society of the ROC, Merck, Organon, and Ferring; attendance fees for expert meetings from Health Promotion Administration, Ministry of Health and Welfare, Taiwan, and the National Science and Technology Council of Taiwan; and support for attending meetings and/or travel from the National Science and Technology Council of Taiwan and Merck. None of the other authors report any conflicts in relation to this work.

**TRIAL REGISTRATION NUMBER:**

N/A.

## Introduction

Infertility is a medical condition experienced by one in six couples globally at some stage during their reproductive years (World Health Organization, 2023). As a recognized medical condition, infertility and many of the associated conditions that come with being infertile require access to quality fertility care services (World Health Organization, 2022). Although in practice this is not always the case and many coverage gaps exist, which likely results in unmet medical need and many people being unable to achieve their desired family size (Fertility Europe, 2023). Surveys often highlight the gap between the number of children couples desire and the numbers they actually have, which can be attributed to a range of cultural and economic factors, but the 17% of couples who are infertile, globally, would likely include many who are unable to access fertility services (World Health Organization, 2023). The inability of infertile couples to receive care due to affordability or other barriers suggests many couples remain involuntarily childless. This unmet need for fertility treatments is unfortunate considering that countries around the world with falling birth rates are seeking to increase fertility rates, and many governments are actively seeking opportunities to encourage couples to have children to combat the effects of aging populations (Chen, 2012; Sobotka *et al.*, 2019).

The traditional burden of disease approaches for evaluating a health impact often look at conditions in isolation, often failing to take into consideration externalities of the condition, i.e. how a condition influences others in society. Economists have established frameworks for evaluating the externalities of economic relationships and how they interact and how health conditions are likely to influence these relationships. In the context of public economics, there are established economic relationships in that working-aged adults work and pay taxes to support older and younger age groups (Mason *et al.*, 2022). As generations age and birth rates have fallen, additional strain is placed on remaining workers who are expected to pay for public programs, e.g. pensions, healthcare, and living allowances for other generations. Infertility is unique as a medical condition as it results in fewer children being born each year resulting in fewer workers in the future available to pay taxes and support public programs. The externality of fewer children results in fewer workers in the future, which places added strain on those remaining workers, often resulting in higher taxes. The externality between birth rates today and higher taxes paid in the future by remaining workers was highlighted by a study from the Bank of Italy in order to draw attention to aging populations and low birth rates (Rizza and Tommasino, 2010). This would suggest the effects of infertility are not isolated to infertile couples and illustrate that all members of society now and in the future can be impacted by low birth rates and infertility (Connolly *et al.*, 2023).

In July 2021, the Taiwanese government introduced a revised subsidy scheme to support all infertile couples seeking medical treatments (Ministry of Health and Welfare, 2022a). The new subsidy for assisted reproduction included NT$100 000 for the first stimulation cycle and NT$60 000 for each additional cycle up to a maximum of six cycles for a woman under the age of 39 and up to three cycles for those aged between 40 and 44. Remaining treatment costs are paid out of pocket by couples. This policy change was partially implemented as part of the Taiwanese government’s family-friendly policies and the desire for more babies (Executive Yuan, 2022), but also with the aim of reducing multiple birth rates by helping to pay for in-vitro fertilization (IVF). The new subsidy policy contributed to an increased number of IVF/intracytoplasmic sperm injection (ICSI) cycles of 31.5% from 2020 to 2021, with an 18.9% increase in live births compared with the year prior to the subsidy (Health Promotion Administration, Ministry of Health and Welfare, 2023). However, it is important to note that there has been increasing use of IVF over the previous years, and other factors could be contributing to increased utilization. Using IVF as a tool to influence birth rates has empirical evidence based on previous studies demonstrating how changes to subsidy schemes can influence numbers of treatment cycles and resulting births (Hsu *et al.*, 2018; Bissonnette *et al.*, 2019).

Evidence in policy making is often lacking at the point of implementation, as governments are often hoping for a desired effect in relation to a new policy. Post-hoc policy evaluation can help to establish the merits of government policy and seek opportunities for adjusting the policy and establishing good governance (OECD, 2020). With publicly available data on the numbers of live births since the introduction of the IVF subsidy policy in 2021 and published data from the Taiwanese government on expenditure, we are in a unique position to evaluate whether the IVF subsidy policy represented a good use of government funds. In our assessment, we evaluate whether the public investment costs associated with a recent subsidy change in Taiwan and the resulting births represent good fiscal policy for government (UDN, 2023). We made the assessment applying an established government perspective framework that accounts for future government costs (e.g. healthcare, education, pensions, allowances, and social benefits) and future taxes and social insurance paid by the cohort of children resulting from this recent subsidy change (Auerbach *et al.*, 1994; Connolly *et al.*, 2017).

## Materials and methods

To evaluate the impact of recent IVF subsidy changes in Taiwan, we applied a generational accounting framework used by governments to evaluate future costs and benefits attributed to policy changes (Auerbach *et al.*, 1994). As the aim of the IVF subsidy increase was to influence the number of births, we estimate the future gross and net tax contributions that will arise in relation to the cohort of IVF-conceived children born as a result of the policy change (Ministry of Health and Welfare, 2022a). A similar modeling approach has been used to evaluate Taiwan’s National Pension Program (Hsieh and Tung, 2016). The analysis described here applies a lifetime assessment of estimated future taxes paid by individuals and age-related government benefits (e.g. pensions, allowances, education) received using age-specific national averages. This required modeling of average age-specific annual incomes adjusted for annual work activity rates and adjusted for mortality.

### Estimating wages and taxes

Data on annual earnings and taxes paid were obtained from The Survey of Family Income and Expenditure (National Statistics, 2021b), which reports age-specific data that were, in turn, used for modeling lifetime taxable income. The average age-specific earnings and taxes paid were adjusted for those working using National Statistics reporting on labor force participation by age (National Statistics, 2021a). These numbers were compared with average tax burden by age reported by the tax authority to validate the amount of taxes paid (Fiscal Information Agency, 2022). Additionally, social insurances, deducted from earnings separately and paid by employer and employee in varying proportions, were included as reported in Table 1. To account for taxes paid in relation to consumption, we applied the VAT rate to age-specific disposable income data, thus estimating lifetime sales taxes paid by individuals (Ministry of Finance, 2017; Directorate General of Budget, Accounting and Statistics, 2021).

**Table 1.** Social insurance contributions paid by employers and employees in Taiwan.

	Total premium	Employee contribution	Employer/employee contribution	Government contribution	Source
Labor insurance	11.00%	2.20%	7.70%	1.10%	Bureau of Labor Insurance (2023)
Employment insurance	1.00%	0.20%	0.70%	0.10%	Bureau of Labor Insurance (2023)
National Health Insurance (NHI)	5.17%	1.55%	3.10%	0.52%	Ministry of Health and Welfare (2022a)

### Public programs paid by government

The analysis described here considers costs that arise from a range of government programs. Firstly, we include per-student costs paid by the government for the duration of education. These costs are adjusted every year of education for the proportion of students attending each level of schooling and are inflated over time (Ministry of Education, 2022). We derive annual average per capita age-specific healthcare expenditure reported by the National Health Insurance for the year 2021 and adjusted for inflation over the analytic time horizon of the model (Ministry of Health and Welfare, 2022a). Furthermore, a range of public assistance programs are available for people in Taiwan based on need at various stages of life and circumstances. These findings are reported in the Survey of Family Income and Expenditure and have been included in our analysis based on the average annual cost per person by age (National Statistics, 2021b).

To account for wage growth, we apply the average economic growth observed between 2013 and 2020, estimated to be 2.78% (National Statistics, 2023). Future government payments, including pensions, are inflated at 1.12% as per consumer price index observed in the period 2012–2022 (Ministry of Labor, 2023). To calculate the present value of future tax and social benefits, a discount rate of 3.5% is applied annually to all costs and tax revenue throughout the life of the IVF-conceived children, which is consistent with similar analyses in Taiwan and consistent with cost–benefit analyses of investment programs by government (Hsieh and Tung, 2016; HM Treasury, 2022). A range of public support programs are available in Taiwan and have been introduced to support families. In our assessment, we include the one-off ‘baby bonus’ payments made by the government. Additionally, we include special allowances for parental leave paid (Bureau of Labor Insurance, 2023) and for monthly childcare services for up to 72 months after delivery (Executive Yuan, 2023).

### Subsidy policy for assisted reproduction

The newly introduced policy provides NT$100 000 for the first fresh cycle and NT$60 000 for each additional cycle up to six cycles of IVF or ICSI (Ministry of Health and Welfare, 2022b). Intrauterine insemination (IUI) is not covered by the current policy. The new subsidy covers approximately 45–62% of the costs of a fresh cycle, with remaining costs paid by couples that are not considered in our public economic assessment. The time period for our analysis captures the numbers of live births for those couples receiving government treatment subsidies between the period March 2022 and July 2023 (16 months) since the introduction of the new policy. Communication from the government in July 2023 reports spending NT$3.8 billion on IVF and ICSI with 11 636 children being born, suggesting a government cost per baby of NT$332 000 (Ministry of Health and Welfare, 2022b; Health Protection Agency, Ministry of Health, 2023). In this regard, the reported births and contributions from government correspond to the same 16-month time period. Furthermore, it is important to note that the government data only reports those couples receiving the public subsidy and does not include those couples treated privately; hence, live births reported here do not correspond with annual reporting of data on cycles in Taiwan. The cost per baby is used as the investment case in our analysis (Ministry of Health and Welfare, 2022b). The average cost per live birth can be influenced by the age of couple and prognosis; therefore, we explore how increasing the cost per live birth influences the investment case for government.

### Analytic outputs

The model annually estimates an average individual’s income and adjusts it for mortality and wage inflation. Analogously, mortality-adjusted annual gross tax and transfers are estimated accounting for wage inflation and cost inflation, respectively. Subsequently, the present value of lifetime fiscal outcomes is estimated (Equations 1–3) for the cohort of live births produced by IVF. These lifetime fiscal outcomes are, in turn, combined to estimate net tax and return on investment (ROI) under different assumptions.


(1) $$\text{Gross}\;\text{income}=N_{0}\times \sum_{t=0}^{101}S(t)\times \frac{Y\left(t\right)+\text{DY}\left(t\right)}{{\left(1+r\right)}^{t}}\times e^{Wi\times t}$$

(2) $$\text{Gross}\;\text{tax}=N_{0}\times \sum_{t=0}^{101}S(t)\times \frac{\text{Tax}\left(t\right)+\text{DY}\left(t\right)\times \text{VAT}\left(t\right)}{{\left(1+r\right)}^{t}}\times e^{Wi\times t}$$

(3) $$\text{Transfers}=N_{0}\times \sum_{t=0}^{101}\sum_{j}^{3}S(t)\times \frac{{\text{TR}}_{j}\left(t\right)\times {\text{pTR}}_{j}\;(t)}{{\left(1+r\right)}^{t}}\times e^{{\text{CPI}}_{j}\times t}$$

Net tax=gross tax – transfers – CLB

$${\text{ROI}}_{\text{gross}\;\text{tax}}=\text{gross}\;\text{tax}\;÷\;\text{CLB}$$

$${\text{ROI}}_{\text{net}\;\text{tax}}=\text{net}\;\text{tax}\;÷\;\text{CLB}$$

where CLB: cost per live birth; CPI_*j*_: cost inflation for transfer *j*; DY(*t*): disposable income, year of age *t*; Gross income: present value of lifetime income; Gross tax: present value of lifetime tax revenue; *j*: type of transfer—(1) healthcare costs, (2) educational costs, (3) other transfers (birth bonus, childcare, and daycare allowance); *N*_0_: number of live births; pTR_*j*_(*t*): proportion receiving transfer *j*, year of age *t*; TR_*j*_(*t*): transfers received by year of age *t*; *r*: discount rate; *t*: year of age; Tax(*t*): direct tax, year of age *t*; *S*(*t*): probability of surviving, year of age *t*; Transfers: present value of lifetime transfers; VAT(*t*): effective VAT rate, year of age *t* (used as a proxy of indirect tax); Wi: wage inflation; Y(*t*): total income, year of age *t*.

This study was reviewed by the Research Ethics Committee D, National Taiwan University Hospital 7, Chung-Shan South Road, Taipei, Taiwan 100, R.O.C. and was determined to be exempt from review. The IRB case number is NTUH-REC No. 202312033W.

## Results

The investment case for IVF in Taiwan using the average cost per life year paid by government results in discounted net taxes of NT$1.55 million over the lifetime of the child. This is based on discounted gross tax revenues of NT$7.26 million after deducting NT$5.37 million in discounted future benefits and the costs for treatment of NT$0.33 million (Table 2). Based on the 11 636 children born since introducing the new subsidy scheme, we estimate future lifetime net tax gains of NT$18.1 billion and gross tax revenues of NT$84.4 billion over the lifetime of the IVF-conceived cohort. The fiscal ROI for government was estimated to be 568% and 2187% over the lifetime of the analysis based on net taxes and gross taxes, respectively.

**Table 2.** Lifetime taxes paid by and transfers received by individuals naturally conceived and conceived by assisted reproduction in New Taiwan Dollars (NT$).

Outcomes	Natural conception (NT$)	IVF conception (NT$)
**Lifetime earnings**	31 683 612	31 683 612
**Gross tax**	7 257 438	7 257 438
Direct tax	769 484	769 484
Indirect tax	1 331 997	1 331 997
National insurance	5 155 957	5 155 957
**Total transfers**	5 373 730	5 373 730
Government transfers	1 982 697	1 982 697
Healthcare costs	874 667	874 667
Educational costs	2 516 366	2 516 366
**Net tax per child**	1 883 708	1 883 708
**Fiscal results (NT$)**		
**Total transfers (−)**	−5 373 730	−5 373 730
**Gross tax (+)**	7 257 438	7 257 438
**Investment (−) per live birth**	N.A.	−331 918
**Net tax (±)**	1 883 708	1 551 789
**IVF ROI based on net tax**	–	568%
**IVF ROI based on gross tax (excludes transfers)**	–	2187%

**Figure 1.**
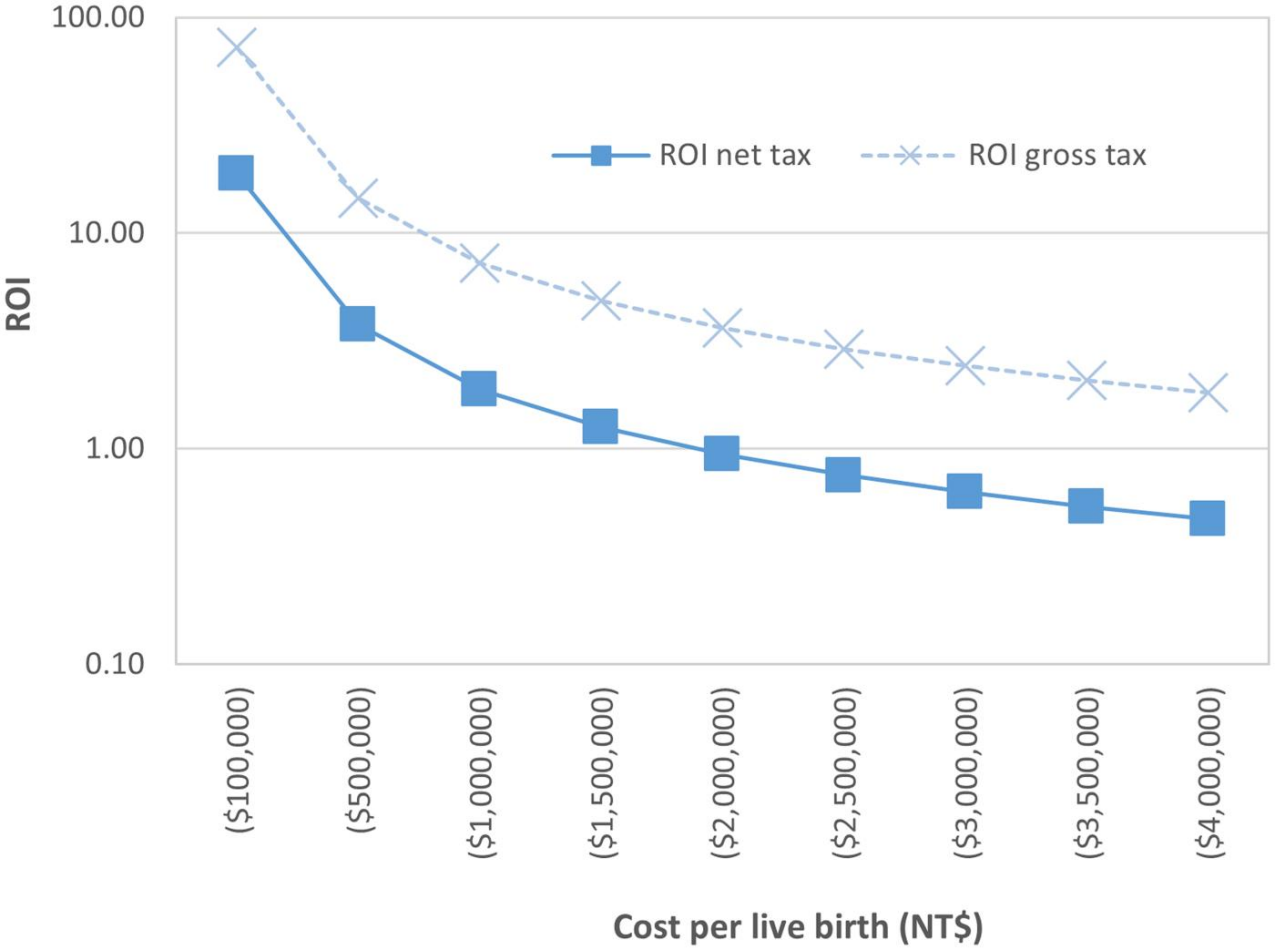
Threshold analysis to identify the fiscal neutrality point, i.e. return on investment (ROI) = 1.0, for an IVF subsidy.

A threshold analysis was conducted to assess at what price per live birth the government can pay and still achieve a positive ROI. The findings are reported for gross and net tax revenues based on varying the cost per live birth (shown as negative values). The point at which the curve crosses 1.0, i.e. fiscal neutrality, is at approximately NT$1.7 million per live birth. In contrast, the threshold analysis for lifetime gross tax value remains >1.0 even when a cost per live birth reaches over NT$4.0 million (Fig. 1).

## Discussion

Applying public economic analytic frameworks to evaluate government programs is a useful tool for assessing long-term costs and consequences likely to impact government accounts. In the context of assisted reproduction, it is a useful tool to evaluate lifetime transactions that children born from reproductive technologies are likely to have with their government in relation to future taxes and public benefits received (Connolly *et al.*, 2009). As shown here, we demonstrate that the 2021 subsidy change in Taiwan is likely to yield positive fiscal gains 5.7 times the initial public investment based on future discount net tax revenues (i.e. lifetime taxes − lifetime public benefits). When viewed in terms of lifetime gross taxes paid, the fiscal yield would be 21.8 times the initial investment. The analysis described here applies a very narrow focus on taxes and government costs. In reality, the gains are likely to be much greater if one considers the financial interactions between generations creating demand and supply for other goods and services further stimulating economic growth to which the government would benefit.

Taiwan, like many advanced economies, is currently facing a demographic challenge that is likely to slow economic growth as birth rates have fallen over the past several decades (Salgado, 2017; Chen, 2023). The primary factor contributing to the declining birth rates in Taiwan is the trend of late marriage and delayed childbirth. The decreasing number of marriages, coupled with delayed age at marriage, has led to an increase in infertility cases, consequently lowering the overall fertility rate (Cheng and Kolk, 2021; Cheng, 2023). The aging of populations has been described as a demographic tax on growth that is likely to reduce economic growth by 0.5–1.0% annually (Salgado, 2017). While having children is a choice that couples should make freely and independently, it is well recognized that without managing this transition, the rapid decline in birth rates will pose economic and fiscal challenges the likes of which the world has never experienced. Many governments facing demographic challenges have expressed interest in a range of family-friendly policy options to lessen the economic strains of raising children, in some cases spending up to 3–4% of GDP on family policies (Sobotka *et al.*, 2019). In response, some governments have recently started to embrace assisted reproduction as one of the myriad of solutions for addressing falling births by increasing public funding for infertility couples (BBC, 2020; Magramo, 2023). This might appear to be a bold policy move; however, the costs of funding IVF are likely to be much lower than the costs that many countries currently spend on existing family-friendly policies (Stone, 2020). Perhaps more importantly, the contribution from assisted reproduction to national births every year is on average 3.5% of all births in Europe, which is greater than the effects of most family-friendly policies currently being used by many countries (Wyns *et al.*, 2022). From a policy perspective, increasing access to fertility treatments can achieve multiple goals, including family formation and increasing birth rates.

The Taiwan government rolled out the first IVF subsidy in 2015, but this was only limited to couples with low income. The subsidy scheme was for one application per year with up to NTD$150 000 per time. Furthermore, only a few IVF centers were involved in this program, as it did not apply to all IVF centers. Due to the limitations mentioned above, only around 140 applications in total came from the rollout of the previous IVF subsidy policy. The new subsidy was rolled out in 2021. Recent policy changes in Taiwan were introduced to reduce the financial barriers associated with seeking treatment for infertile couples. This policy change was partially introduced due to the positive pronatalist environment in Taiwan, and reflects earlier calls for strengthening reproductive health services as one of the measures for increasing birth rates (Chen, 2012). For many years, the Taiwanese government has pursued a pronatalist agenda to encourage family building and births (Yip and Chen, 2016). The new subsidy policy in 2021 was done not only in recognition of the impact of being infertile but also as a measure to influence birth rates. The effects of assisted reproduction can be small but important as observed in other Asian countries that have adopted funding of IVF as part of a pronatalist agenda (Tan, 2020). While assisted reproduction can play a part of the policy mix for influencing births, a range of options should be pursued to influence birth rates, including fertility education, increasing marriage rates, and supporting first births (Yip and Chen, 2016).

In Taiwan, the government’s subsidies have encouraged couples planning to undergo assisted reproductive treatments to start the process earlier, thereby increasing the willingness of infertile couples to pursue such treatments. However, it is essential to note that this study has its limitations. The policy was implemented in 2021, and the calculated costs may reflect the initial stages of policy execution, potentially including more new patients and those who successfully conceived in the first or second treatment cycles. Consequently, the live birth rate appears higher in the current statistics that result in the estimated cost for a successful live birth of around NT$332 000 (Ministry of Health and Welfare, 2022b). However, it is foreseeable that as the subsidy program progresses, the number of treatment cycles for difficult cases with multiple failures may increase, leading to a decline in the live birth rate and an increase in government subsidy costs. Additionally, the cost per live birth is estimated from aggregate figures provided by the Taiwanese government for total spending on assisted reproduction from the new policy, and the resulting number of children born 11 636 during the new policy period. Within these figures exists a heterogenous population defined by age and etiology, which could influence the live birth rate and the aggregate cost per live birth. For example, younger couples could conceive after one treatment cycle while others might require four or five cycles before conceiving and having a live birth, which influences the cost per live birth across a heterogenous population. To account for likely variation, we performed a sensitivity analysis in which the average cost per live birth varies in order to estimate the maximum the government should be willing to spend and still remain fiscally neutral. In other words, the investment ROI is >1.0, meaning the government receives an amount in future net taxes that is greater than the costs per live birth of the child. The threshold analysis indicates that spending up to approximately NT$1 700 000 per live birth is still fiscally positive. This value is more than five times the current average spending per live birth. From the government perspective, the optimal outcome is to pay less per live birth rather than paying more. This would suggest that early identification, removal of barriers to care, and early access to treatment can offer greater fiscal returns for the government. For future research, it may be beneficial to explore subsidies for different age groups, considering the varying economic outcomes resulting from differences in live birth rates, especially for women older and younger than 40 years, as well as to lessen the limitation of embryo transfer numbers for women with advanced age. Therefore, the analysis can provide more targeted and efficient planning for the government’s subsidy policy in terms of eligible recipients and cost allocation.

The results presented here suggest that investment costs in IVF can yield significant gains for the government in future net tax revenue. The investment case described here is based on the average cost per live birth. What is not known from our analysis is the degree to which this policy can be increased, and at what point there become diminishing marginal returns from the investment. There is likely to be a point at which additional funding for fertility treatments has no effect and the investment yield decreases. To protect against this and the oversupply of services, policies often apply restrictions to the number of cycles and co-payments, which prevents moral hazard, i.e. situations where doctors and patients may overuse medical procedures as they do not face the financial risks associated with consumption. For policymakers, it is important to have the right incentives and limits to protect against over-consumption, which can also be physically hazardous for patients, but cautioning not to create barriers that inhibit people from accessing the care they need. This is not only true for assisted reproduction but for all medical services provided by national health systems.

Economists have long considered the value of children to households and society, which can offer many insights on how to evaluate IVF policy. For households, there can be limited economic rewards associated with children in the short term due to the costs of raising children (Lino, 2020). Governments are aware of these costs and often introduce family-friendly policies in order to alleviate the costs of raising children with programs that include subsidized childcare, tax breaks, and other state-sponsored allowances for raising children. The intent of these policies is not necessarily a selfless act on the part of government to promote family building, as the benefits of future children to government are well established based on the future net taxes that these children generate. A range of studies illustrate the value that children bring for government and for maintaining economic growth for government. Due to concerns over population aging and falling birth rates, there has been increasing interest in pronatalist policies that more actively seek to encourage childbirth (Sobotka *et al.*, 2019).

Several weaknesses should be considered when interpreting the results described here. Firstly, like all government projections, it is necessary to make projections about the future, which may not come to fruition. One of the main factors influencing our analysis is projections of future wage growth. In our analysis, we applied a rate of 2.78% based on the economic growth rate observed between 2013 and 2020. By using this period, we have avoided the high wage growth rates currently observed during the post-pandemic period. As this rate is applied consistently throughout the model, there is the possibility of overestimating wages, which influences the taxes paid. This is particularly relevant as considerable uncertainty exists regarding how aging societies might influence wage growth in the future where wages might fall or stagnate (Maestas *et al.*, 2016). We have applied a discount rate of 3.5%, which is recommended by some leading experts on government perspective cost–benefit analysis. However, there are plenty of people who might suggest a higher discount rate is more appropriate. We are of the view that the future value of money is best reflected by the long-term bond rate in a country. The current bond rate in Taiwan is between 1% and 2%, suggesting that our discount rate is conservative (National Treasury Administration, 2023). Additionally, in our analysis, we do not evaluate specific procedures related to assisted reproduction and use the aggregate number of births to determine the cost per live birth funded by the government. Any changes in the live birth rate that positively or negatively affect the success rates achieved in clinics could influence the cost per live birth and the results described here.

## Conclusions

The new assisted reproduction subsidy scheme introduced in Taiwan in 2021 has generated a meaningful number of live births with the prospect of generating long-term fiscal value for government. The fiscal ROI described here indicates that the Taiwanese government receives increased net tax revenue resulting from the policy that is 21 times in terms of the lifetime gross taxes paid and five times more in future net tax revenue than the costs of the fertility subsidy policy per live birth. Improved subsidies and access to fertility treatments is not a panacea for addressing falling birth rates, but it does help to support those couples seeking to have children and should be seen as one of the many options that governments can consider in relation to addressing falling birth rates.

## Data Availability

The data on which the analysis is based was derived from published materials. All material used has been referenced appropriately to enable readers to identify sources of information.
